# Structural Basis for Differential Neutralization of Ebolaviruses

**DOI:** 10.3390/v4040447

**Published:** 2012-04-05

**Authors:** Shridhar Bale, Joao M. Dias, Marnie L. Fusco, Takao Hashiguchi, Anthony C. Wong, Tong Liu, Ana I. Keuhne, Sheng Li, Virgil L. Woods, Kartik Chandran, John M. Dye, Erica Ollmann Saphire

**Affiliations:** 1 Dept. of Immunology and Microbial Science, The Scripps Research Institute, La Jolla, CA 92037, USA; Email: sbale@scripps.edu (S.B.); jmcndias@yahoo.com (J.M.D.); mhavert@scripps.edu (M.L.F.); takaoh@scripps.edu (T.H.); 2 Dept. of Microbiology and Immunology, Albert Einstein College of Medicine, Bronx, NY 10461, USA; Email: anthony.wong@med.einstein.yu.edu (A.C.W.); kartik.chandran@einstein.yu.edu (K.C.); 3 Dept. of Medicine, University of California San Diego, La Jolla, CA 92093, USA; Email: toliu@ucsd.edu (T.L.); s4li@ucsd.edu (S.L.); vwoods@ucsd.edu (V.L.W.); 4 Virology Division, United States Army Medical Research Institute of Infectious Diseases, Ft. Detrick, MD 21702, USA; Email: ana.kuehne@us.army.mil (A.I.K.); john.m.dye1@us.army.mil (J.M.D.); 5 The Skaggs Institute for Chemical Biology, The Scripps Research Institute, La Jolla, CA 92037, USA

**Keywords:** Filovirus, Ebola, ebolavirus, Sudan virus, neutralization: glycoprotein, antibodies, structure

## Abstract

There are five antigenically distinct ebolaviruses that cause hemorrhagic fever in humans or non-human primates (Ebola virus, Sudan virus, Reston virus, Taï Forest virus, and Bundibugyo virus). The small handful of antibodies known to neutralize the ebolaviruses bind to the surface glycoprotein termed GP_1,2_. Curiously, some antibodies against them are known to neutralize *in vitro* but not protect *in vivo*, whereas other antibodies are known to protect animal models *in vivo*, but not neutralize *in vitro*. A detailed understanding of what constitutes a neutralizing and/or protective antibody response is critical for development of novel therapeutic strategies. Here, we show that paradoxically, a lower affinity antibody with restricted access to its epitope confers better neutralization than a higher affinity antibody against a similar epitope, suggesting that either subtle differences in epitope, or different characteristics of the GP_1,2_ molecules themselves, confer differential neutralization susceptibility. Here, we also report the crystal structure of trimeric, prefusion GP_1,2_ from the original 1976 Boniface variant of Sudan virus complexed with 16F6, the first antibody known to neutralize Sudan virus, and compare the structure to that of Sudan virus, variant Gulu. We discuss new structural details of the GP_1_-GP_2_ clamp, thermal motion of various regions in GP_1,2_ across the two viruses visualized, details of differential interaction of the crystallized neutralizing antibodies, and their relevance for virus neutralization.

## 1. Introduction

Ebolaviruses are filamentous viruses with a negative-sense RNA genome that cause severe hemorrhagic fever with high lethality. There are five different ebolaviruses, each named after the location of the outbreak in which it was first discovered: Ebola virus (EBOV; formerly known as Zaire ebolavirus), Sudan virus (SUDV), Reston virus (RESTV), Taï Forest virus (TAFV), and Bundibugyo virus (BDBV) [1,2]. These virions differ by 40-50% in primary amino acid sequence and are antigenically distinct. Sudan virus was the first ebolavirus to be discovered when it caused an outbreak of hemorrhagic fever among cotton factory workers in Nzara, Sudan, in 1976 [3]. Travel of these patients and their caretakers spread the disease to other villages including Maridi, where in the hospital at which several of the patients were treated, the doctor-in-charge and 61 of the 154 nursing staff became infected [3]. The variant of Sudan virus linked to this outbreak is termed Boniface (or SUDV-Bon) after the index case. This emergence of SUDV-Bon ultimately led to 284 cases of disease and 151 deaths. 

A separate variant, termed Gulu (or SUDV-Gul), is a modern variant linked to a disease outbreak in the Gulu municipality of Uganda in 2000 [4]. The Gulu outbreak was the largest outbreak of Ebola virus disease yet recorded, with 425 cases and 224 deaths and a large number of cases among children and adolescents. The glycoprotein (GP_1,2_) of SUDV-Bon and SUDV-Gul differ in sequence by ~5% and an ideal diagnostic or immunotherapeutic for Sudan virus would be able to recognize and neutralize both the Boniface and Gulu variants. An even better diagnostic or therapeutic would be able to recognize all ebolaviruses, taking advantage of structural information of the distinct GP_1,2_ glycoproteins of each virus.

The trimeric, membrane-attached glycoprotein GP_1,2_ is the only virally encoded protein present on the virus surface and is critical for virus adhesion, entry and internalization into target cells. In infected cells, GP_1,2_ is cleaved by furin to yield two subunits, termed GP_1_ and GP_2_, that remain linked by a disulfide bond. GP_1_ is responsible for recognition and engagement of new target cells, while GP_2_ drives fusion of the viral membrane with the endosomal membrane of the target cell. Antibodies against EBOV GP_1,2_ have been tested for protection against EBOV challenge in various models with mixed outcomes [5,6,7,8,9,10]; although recent studies are quite promising [11].

A crystal structure of trimeric GP_1,2_ from Ebola virus variant Mayinga (EBOV-May) in its prefusion form (PDB code 3CSY) [12] revealed that the three GP_1_ subunits are gathered and tethered into a bowl/chalice shape in the viral surface trimer by the three GP_2_ subunits that wrap around the GP_1_s. In crystal structures of the post-fusion six-helix bundle conformation of GP_2_, heptad repeat 1 (HR1) adopts a single long helix [13,14,15]. However, in the prefusion structure, the hydrophobic fusion peptides of GP_2_ (residues 529-535) wrap around the outside of the trimer, packing against the GP_1_ subunits of adjacent monomers, and only the first of two heptad repeats in GP_2_ is visible. This single helix is broken into four separate sections in the prefusion structure: HR1_A-D_. The crystal structure also illustrated that within GP_1_, putative receptor-binding regions are somewhat sequestered inside the bowl of the chalice at the top of GP_1,2_. A subsequent crystal structure of SUDV-Gul GP_1,2_ (PDB code 3S88) [16] illustrates that its GP_1,2_ has a similar fold but curiously, different electrostatic characteristics than EBOV-May GP_1,2_. EBOV-May GP_1,2_ is neutral/basic while SUDV-Gul GP_1,2_ is acidic at the base where the GP_1_ and GP_2_ subunits meet. In addition, the ectodomain of SUDV GP_1,2_ is more susceptible to proteolysis than EBOV GP_1,2_. Treatment of SUDV GP_1,2_ with either thermolysin or cathepsin L/B results in significant degradation of GP_1,2_ [17]. It is possible that the different electrostatics may contribute to the differing behavior of the GP_1,2 _in the endosome [16]. Recent studies also show that the GP_1,2 _of filoviruses exhibit distinct protease preferences. The GP_1,2_ of EBOV is strongly dependent on the endosomal protease cathepsin B while the GP_1,2_ of SUDV is not dependent on the protease [18,19].

Both EBOV-May GP_1,2_ and SUDV-Gul GP_1,2_ structures were determined in complex with antibodies. EBOV-May GP_1,2_ was crystallized in complex with an EBOV-specific human antibody termed KZ52, which was derived from a human survivor of a natural infection in Kikwit, Zaire (EBOV-May), in 1995 [5]. SUDV-Gul GP_1,2_ was crystallized in complex with a novel Fab termed 16F6 that was raised by immunization of mice with irradiated SUDV-Bon virions [16]. Unexpectedly, the epitopes of KZ52 and 16F6 overlap, at the base of the GP_1,2_ spike where GP_2_ meets GP_1_[16], suggesting that the two antibodies may function in the same way and that the shared site may be a hot spot for neutralization of ebolaviruses.

Here we report new molecular details of neutralization of the different GP_1,2_s by respective antibodies. Surface Plasmon Resonance and neutralization assays reveal that paradoxically, the antibody with lower affinity for recombinant GP_1,2_, with hindered access to GP_1,2_ epitope, has better neutralizing capacity. Here, we also report a new crystal structure, to 3.35 Å resolution, of SUDV-Bon GP_1,2_ from the historical 1976 Boniface variant, in complex with mAb 16F6. This SUDV-Bon GP_1,2_ structure allows better visualization of key contacts between GP_1_ and GP_2_ and the “chain reversal regions” of GP_2_ involved in conformational changes during fusion (a step likely blocked by both 16F6 and KZ52) [20]. Comparison of the now three available structures of ebolavirus GP_1,2_s (SUDV-Bon, SUDV-Gul and EBOV-May) provides new insights into particular components of the two neutralizing epitopes and the puzzle of what constitutes an effective neutralizing antibody response against ebolaviruses.

## 2. Results and Discussion

### 2.1. Crystal Structure of Sudan Virus Boniface GP1,2

**Figure 1:**
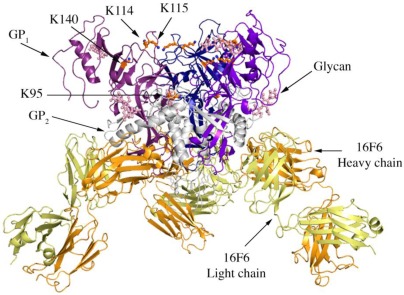
Cartoon representation of trimer of SUDV-Bon GP_1,2_ bound to 16F6. The GP_1_ chains are colored in various shades of blue and GP_2_ chains are colored in white. The light chain of 16F6 is shown in pale yellow and the heavy chain of 16F6 is colored bright orange. The sugar residues on the glycoprotein are shown in ball and colored cyan. Lysine residues critical for receptor binding are shown in ball and stick and colored orange.

The crystal structure of SUDV-Bon GP_1,2_ bound to antibody 16F6 was determined to a resolution of 3.35 Å using molecular replacement. The crystallographic asymmetric unit contains one GP_1,2_ monomer bound to one 16F6 Fab fragment. The final model contains residues 32-191, 213-285, 300-311 and 510-614 of GP_1,2_ and residues 1-220 and 1-212 of the heavy and light chains of 16F6, respectively. A disulfide bond between C53 and C609 covalently links the GP_1_ and GP_2_ subunits together, and is only visible in structures of SUDV, but not EBOV GP_1,2_. In all three ebolavirus GP_1,2_ structures, GP_1_ and GP_2_ bury a surface area of ~2400 Å^2 ^on each other and are held together by numerous hydrogen bonds and non-bonded contacts. Although GP_1,2_ was treated with peptide-N-glycosidase F (PNGase F), clear electron density is still observed for the first two monosaccharides of glycans attached to N257 (NAG 850) in GP_1_ and N563 (NAG 901) in GP_2_ indicating that these sites (in both SUDV and EBOV GP_1,2_) are resistant to PNGaseF digestion (Figure 1). The trimeric form of the GP_1,2_ is obtained by application of 3-fold crystal symmetry, and has an approximate dimension of 95 Å × 95 Å × 90 Å.

Four lysines (95, 114, 115, and 140) in the putative receptor-binding head of GP_1_ have been identified as critical for attachment of EBOV to target cells [21]. Three of these (114, 115, and 140) are strictly conserved among all known ebolaviruses, while position 95 is occupied by glutamine in some variants of SUDV (but is lysine in the SUDV-Bon and SUDV-Gul sequences crystallized). In all three ebolavirus GP_1,2_ structures, K95 is buried deep in the chalice bowl of GP_1_ and projects into GP_2_ rather than out toward the target cell and receptor (Figure 1). Inside GP_1,2_, K95 forms a hydrogen bond with the main-chain carbonyl of T576 and forms van der Waals interactions with L573, which is located in the loop between HR1_B_ and HR1_C_ of GP_2_. Hence the crystal structures support biochemical predictions [21] that the observed functional importance of K95 may not be in direct receptor engagement, but rather in proper maintenance or springing of the prefusion GP_1,2_ assembly. Alternatively, K95 may become better exposed for interaction with host factor(s) in the endosome if any conformational changes occur as a result of receptor binding. K115 also points down toward the viral membrane, but is solvent exposed and could potentially reorient upon receptor engagement. K114 and K140 project into solvent towards the target cell and may serve as direct contacts for receptor. 

In the prefusion conformation of ebolavirus GP_1,2_, the first heptad repeat of GP_2_ is broken into four structural segments (HR1_A-D_) that wrap around GP_1_. We observe in all three structures (SUDV-Gul, SUDV-Bon, and EBOV-May) that residues R580-T581 of HR1_C_ form a short β strand that assembles into a continuous β sheet with the K95-S99 β strand of the GP_1_ receptor-binding head. These residues are completely conserved among all ebolaviruses and marburgviruses.

**Figure 2:**
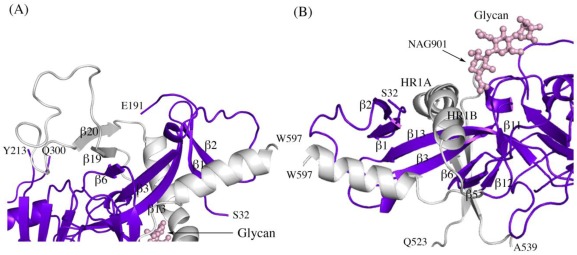
Secondary structure interactions between SUDV-Bon GP_1_ (colored blue and purple) and GP_2_ (colored light grey). **(A)** Close up of the continuous seven-stranded twisted β sheet formed by five β strands of the GP_1_ base (β2, β1, β13, β3, and β6 in blue) and two antiparallel β strands of the GP_2_ internal fusion loop (β19 and β20 in grey). **(B)** The first heptad repeat of GP_2_ (which contains the grey coils labeled HR1A and HR1B and the loop afterwards) is clamped by a horseshoe surface formed by five β strands of GP_1_ base (β12, β5, β6, β3, β13), a second small β sheet of the GP_1_ base (β1 and β2) and a glycan (pink ball-and-stick). To orient the reader, key residues are labeled.

Other structural details not previously reported include the noncovalent assembly by which GP_1_ is anchored to GP_2_. Five β strands of the GP_1_ base and two antiparallel β strands of the GP_2_ internal fusion loop combine to form a continuous seven-stranded twisted β sheet. The GP_1_ component of that sheet and a second small β sheet of the GP_1_ base together assemble a semicircular GP_1_ horseshoe surface that clamps the first heptad repeat of GP_2_ in its metastable, prefusion structure (Figure 2 A,B). In order for GP_2_ to rearrange into its post-fusion six-helix bundle structure [13,15] both the continuous GP_1_-GP_2_β sheet and the horseshoe clamp must be broken. The C-terminal heptad repeat 2 is disordered in all three SUDV-Bon, SUDV-Gul and EBOV-May structures, which may result from the functional mobility of this region as well as the lack of the transmembrane regions that tether GP_1,2_ on the viral surface. 

### 2.2. Comparison of SUDV-Bon and SUDV-Gul Glycoproteins

The Boniface and Gulu variants of Sudan GP_1,2_ differ in sequence by ~ 5%. The clones of SUDV-Bon and SUDV-Gulu GP_1,2_ used for crystallization contain 449 residues, of which 7 differ (position 237 in the glycan cap - N/D for Bon/Gul; position 243 in the glycan cap - L/R; position 272 in the glycan cap -K/R; position 310 in the glycan cap - T/A; position 503 near the furin cleavage site - V/T; position 506 near the furin cleavage site -R/K; and position 631 close to the transmembrane region - I/V). Overall, structures of SUDV-Bon and SUDV-Gul GP_1,2_ align with an r.m.s.d. of 1.0 Å, and the entire SUDV GP_1,2_-antibody complexes align with an r.m.s.d. of 0.74 Å, because the Fab components of the complexes are better ordered and better superimpose than the glycoproteins to which they are bound.

### 2.3 Implications for fusion

A CX_6_CC motif in GP_2_ that is conserved among all ebolaviruses is visible in both structures of SUDV GP_1,2_. This motif contains the disulfide bond that anchors GP_1_ to GP_2_ (C53-C609) and a second disulfide bond within GP_2_ (C601-C608). The glycoproteins of some retroviruses have a CXXC motif within the receptor-binding subunit that isomerizes to release the disulfide bond between the receptor-binding and fusion subunits [20]. Filoviruses, however, lack such a motif in GP_1_, and it is currently unknown if the GP_1_-GP_2_ disulfide link, which is encoded by a different motif, is retained or released during fusion. It has been speculated that endosomal thiolreductases could reduce the C53-C609 GP_1_-GP_2_ bond [22], and mild reduction of the enzymatically processed 19 kDa GP_1,2_ does confer binding to liposomes (an ability presumably attained via conformational change, as unreduced 19 kDa GP_1,2_ does not bind liposomes) [23]. The neighboring, C601-C608 intra-GP_2_ disulfide bond however, is observed in pre-fusion as well as post-fusion crystal structures and thus, probably remains intact during fusion. Given the close proximity of both the GP_1_-GP_2_ and GP_2_-GP_2_ disulfide bonds, reduction is either very specific for the GP_1_-GP_2_ disulfide bond or plays no role during fusion. Separate possibilities are that GP_1_ simply rotates out of the way, or that continual enzymatic processing of GP_1_ by cathepsins digests enough of GP_1_ to remove steric hindrance to GP_2_ conformational rearrangement [22]. 

**Figure 3:**
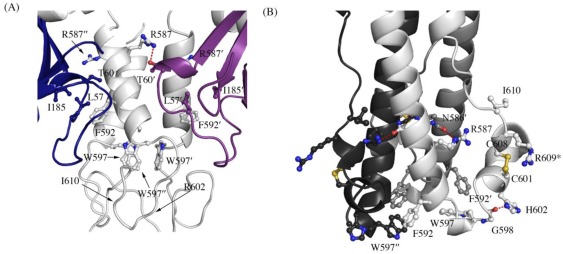
Interaction of residues in the chain reversal region in (A) the prefusion SUDV-Bon GP_1,2_ and (B) the post fusion EBOV-May GP_2_. Prefusion SUDV-Bon is used here as it is better ordered than prefusion EBOV-May. Interacting residues are shown in ball and stick. In (A), different monomers of GP_1_ are colored blue and purple and the three copies of GP_2_ are all colored light grey. In (B), the three copies of GP_2_ are colored different shades of grey. Equivalent residues in the 3-fold related protomers are labeled with ´ and ´´ respectively. Hydrogen bonds are shown as red dashed lines. The residue R609* in the postfusion form is an engineered mutation to replace the cysteine residue (C609) in the native protein that is involved in a disulfide bond with C53 of GP_1_.

A recent study by Delos *et al.* has revealed residues in the “chain reversal region” of GP_2_ critical for fusion of Ebola virions [20]. This region comprises a short stretch of hydrophobic residues, a glycine-glycine pair, a CX_6_CC motif, and a bulky hydrophobic residue after the motif. Mutational analysis in EBOV GP_1,2_ has shown that residues R587, F592, W597, G598, H602, and I610 are critical for viral infectivity. All the above residues except H602 (which is R in SUDV and RESTV) are conserved in SUDV. The better ordered structures of SUDV provide us with newer insights into the role of the conserved residues in the prefusion conformation, and are reported here. Interactions made by the side chains of these residues in the prefusion-form in SUDV and the post-fusion form in analogous EBOV (strain Mayinga, PDB code 1EBO) are shown in Table 1. In the pre-fusion state, the conserved residues make interactions with GP_1_ (hydrogen bonding to T60′ and stacking interaction against L57 and I185 (Figure 3A); the ′ denotes residues from a 3-fold related monomer). W597 is involved in a stacking interaction with other W597 residues from two 3-fold related monomers, suggesting its role in stabilizing the trimeric form in the heptad repeat region. Definitive density was not seen for the side chains of R602 and I610 in either SUDV-Bon or SUDV-Gul GP_1,2_ and we could not assign any interactions of these residues; positions 602 and 610 are thus modeled as alanine. However, in the post-fusion state, the conserved residues make interactions solely with residues in GP_2_ (Figure 3B). In addition, the conserved residues make interactions with different residues in the pre-fusion and post-fusion forms suggesting a greater role of these residues during the fusion process.

### 2.4 Interactions between 16F6 and SUDV GP

**Figure 4:**
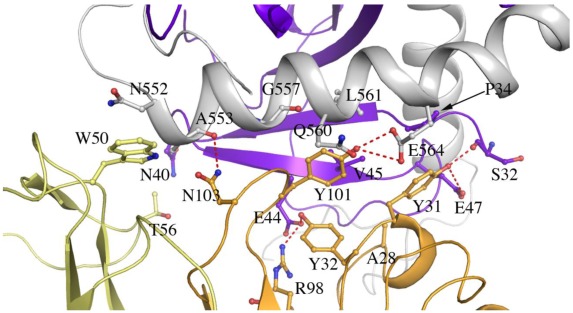
Residues at the interface of SUDV-Bon GP_1,2_ and 16F6 (cutoff distance of 3.5 Å). GP_1_ is colored purple, GP_2_ is colored white, the 16F6 heavy chain is colored orange and the light chain is colored pale yellow. Hydrogen bonds are shown as red dashed lines.

The complementarity determining regions (CDRs) H1 and H3 of 16F6 form a network of hydrogen bonds, van der Waals interactions and one salt bridge to the GP_1_ base. CDR L2 also hydrogen bonds to the GP_1_ base and forms additional hydrophobic interactions to the stem region of the internal fusion loop of GP_2_ (Figure 4). Specific interactions between 16F6 and the glycoprotein have not been previously reported and are shown in Table 2. The heavy chain and light chain of 16F6 bury a surface area of ~1630 Å^2 ^between them. The antibody 16F6 interacts with GP_1,2_ primarily using its heavy chain, burying an area of ~350 Å^2^ with GP_1_ and ~200 Å^2^ with GP_2_. The interface between GP_1,2_ and 16F6 is predominantly hydrophobic with the exception of four hydrogen bonds.

### 2.5 Thermal Motion in GP

**Figure 5:**
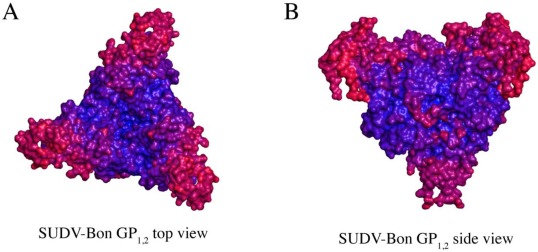
Comparison of thermal factors (B-factors) of various regions of SUDV GP_1,2_ (Panels A and B). Regions of low thermal mobility are deep blue (deep blue color set at ~ 80 Å^2^), whereas regions of high thermal mobility are red (deep red color set at ~ 220 Å^2^). The glycan cap and C-terminal “stem” regions of SUDV GP_1,2_ have higher B-values than the core region, indicating higher motion in those regions.

**Figure 6:**
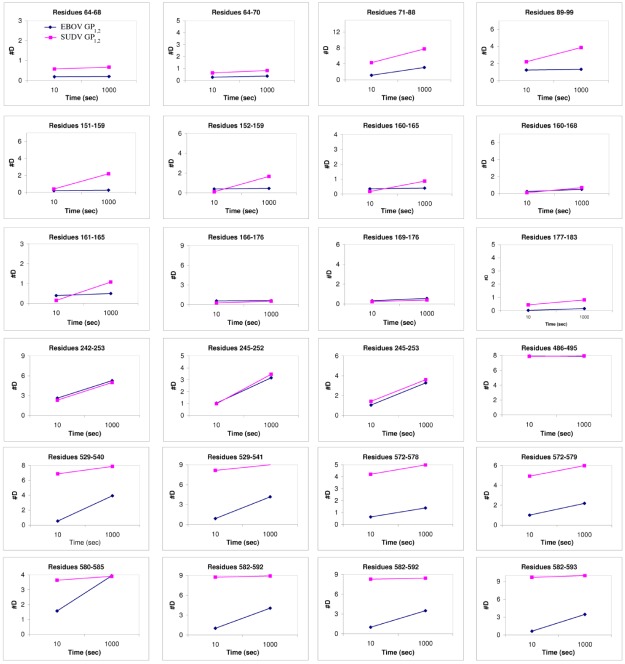
Plots of number of deuterons *vs*. time. These compare the deuteration levels of key peptides of EBOV GP_1,2_ (shown in blue) to their counterparts in SUDV-Gul GP_1,2_ (shown in magenta), measured over a time scale of 10-1000 sec using deuterium exchange mass spectrometry [17].

Comparison of B-factor values (an atomic displacement parameter arising from thermal vibration of atoms and static disorder of atoms in different unit cells of the protein crystal) of key portions of GP_1_ and GP_2_ in SUDV GP_1,2_ reveals that motion predominates in the glycan cap regions, the C-terminal half of the fusion loop, and the visible C-terminal regions of GP_2_ (Figure 5). How does SUDV compare to EBOV GP_1,2_ in this regard? Deuterium Exchange Mass Spectrometry (DXMS) reveals that although GP_1_ of SUDV and EBOV exhibit nearly identical rates of exchange of amide hydrogens with solvent deuterium, all regions of GP_2_ of SUDV, including the fusion loop, heptad repeats, disulfide-containing linker and C-terminal regions, are fundamentally more mobile than those of EBOV GP_1,2_ [17] (Figure 6). Interestingly, the disulfide-containing linker regions of GP_2_ are only visible in crystals of SUDV GP_1,2_, not EBOV GP_1,2_. The unique crystal packing environment of the SUDV I23 unit cell and the acute angle made by the bound 16F6 antibody may have constrained this region.

**Figure 7:**
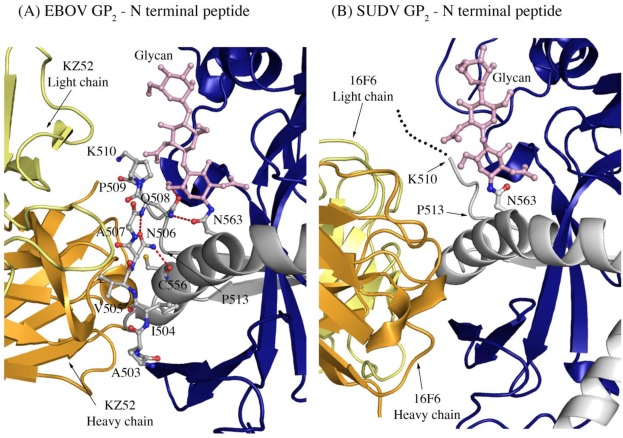
The N-terminal peptide of GP_2_ in (A) EBOV-May GP_1,2_ and (B) SUDV-Bon GP_1,2_. In both panels, GP_2_ is shown in ball-and-stick and colored grey. GP_1_ is colored purple and the heavy and light chains of the antibody are colored bright orange and pale yellow, respectively. Note that the first eight residues of GP_2_ are disordered in SUDV-Bon/-Gul GP_2_ and are shown as black dots. Hydrogen bonds in (A) are shown as dashed red lines. There are no hydrogen bonds observed in (B) as the N-terminal peptide is disordered. The glycan residue connected to N563 is shown in ball and stick and colored light pink.

Another difference in mobility between SUDV and EBOV GP_1,2_ relevant for antibody binding is the N terminus of GP_2_, which is released from GP_1_ by furin cleavage. In EBOV GP_1,2_, this peptide is hydrogen bonded to the GP_1,2_ core, is bound between heavy and light chains in the central region of the KZ52 paratope and forms a significant portion of its epitope. By contrast, in the SUDV structure, the same peptide is disordered and is not bound by 16F6. Instead, 16F6 binds the underlying GP_1,2_ core beneath the peptide (Figure 7). 

One explanation could be that the GP_2_ N terminus is simply tacked down in the EBOV GP_1,2_ crystal structure by KZ52 binding. A more likely explanation, supported by DXMS, is that the mobility of this region fundamentally differs between SUDV and EBOV, even in the absence of antibody binding. Key differences in sequence between SUDV and EBOV at this site may explain this observation. The anchor point of the GP_2_ N terminus to the GP_1,2_ core is residue G509. At position 509, EBOV contains a proline, which may restrict mobility, while SUDV contains a glycine, which may enhance mobility. Further, residues N506 and Q508 of EBOV use both their terminal oxygen and nitrogen atoms to form a network of hydrogen bonds to the EBOV GP_1,2_ core; specifically, to amino acids as well as the attached glycan of heptad repeat 1. By contrast, position 506 is K in SUDV-Gul and Q in SUDV-Bon, and position 508 is T in both SUDV GP_1,2_ (Gulu and Boniface). None of those hydrogen bonds made by EBOV are observed for SUDV, and the specific hydrogen bonds made by the side-chain oxygens of N506 and Q508 in EBOV are not possible for the corresponding residues in SUDV. The key differences in sequence and inherent mobility of N-term region of SUDV GP_1,2_ reflects in a marginally varied epitope for binding of 16F6.

### 2.6 Comparison of Antibody-GP complexes

**Figure 8:**
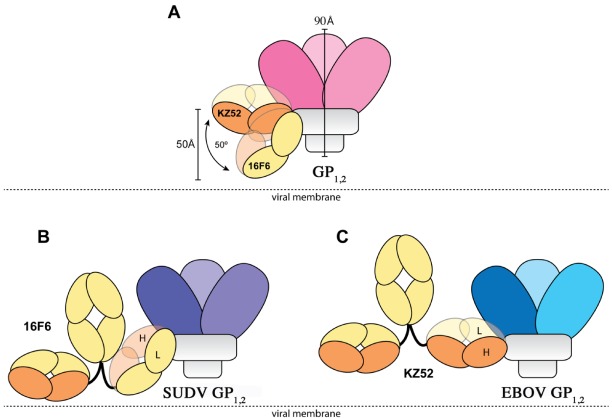
Cartoon illustration of the different modes of antibody binding. **(A)** KZ52 binds perpendicular to the central axis of GP_1,2_ (parallel to the viral membrane), while 16F6 recognizes the overlapping epitope from an acute angle. The central axes of the two Fabs make a 50° angle with each other, and the constant portion of the 16F6 Fab is 50 Å lower than that of KZ52. Fab heavy chains are colored orange, while light chains are colored yellow. Approximate location of the viral membrane is indicated by a dotted line. C terminal portions of GP_2_ that anchor the visualized trimer to the viral membrane are disordered and not drawn here. **(B)** Model of a 16F6 IgG binding SUDV GP_1,2_ (purple) based on the 16F6 Fab-SUDV-Bon GP_1,2_ complex (PDB: 3VE0). **(C)** Model of a KZ52 IgG binding EBOV-May GP_1,2_ (blue) based on the KZ52 Fab-EBOV-May GP_1,2_ complex structure (PDB: 3CSY). The IgG and GP_1,2_ fragments are drawn to scale in the models.

The crystal structures reveal that the 16F6 and KZ52 antibodies bind at overlapping epitopes that contain residues from both GP_1_ and GP_2_. However, the number of interactions made by each antibody with its respective antigen differs (Table 3). 16F6 interacts with SUDV GP_1,2_ via 7 hydrogen bonds and 19 non-bonded contacts. KZ52 interacts with EBOV GP_1,2_ via 13 hydrogen bonds and 29 non-bonded contacts. Hence, the binding of KZ52 to EBOV GP_1,2_ is expected to be stronger than the binding of SUDV GP_1,2_ to 16F6. Accordingly, Surface Plasmon Resonance studies reveal that the K_d_ of KZ52 binding to recombinant, soluble EBOV GP_1,2_ ectodomain is 6.31 ± 3.70 nM while the binding of 16F6 to recombinant, soluble SUDV GP_1,2_ ectodomain is 1.94 ± 1.40 µM to SUDV-Bon, and 1.59 ± 1.0 µM to SUDV-Gul. Further, the angle the antibodies make when binding against the core of the GP_1,2_ differs dramatically. KZ52 binds GP_1,2_ at an angle more parallel to the viral membrane, while 16F6 is angled 50° downward toward the membrane, such that the C-terminal, constant regions of 16F6 are 50 Å closer to the viral membrane than those of KZ52 (Figure 8). The hinge regions attached to the membrane-proximal end of the 16F6 Fab must therefore adopt acute angles so that the other Fab and Fc of the IgG do not penetrate the viral membrane. It is a bit surprising that the 16F6 IgG can reach down to access the epitope from that angle. 

**Figure 9:**
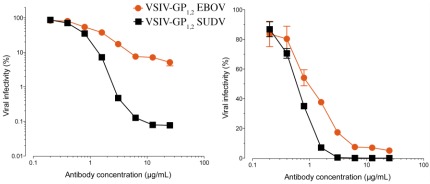
The ability of 16F6 and KZ52 IgGs to neutralize SUDV GP_1,2_-bearing VSIV or EBOV GP_1,2_-bearing VSIV, plotted in a log (A) and linear (B) scales. VSIV particles were pre-incubated with the indicated concentrations of antibody for one hour at room temperature, and then exposed to Vero cells at 37° C. Viral infectivity was scored at 16 hours post-infection. The starting titers were 9×10^7^ IU/ml for EBOV and 3×10^7^ IU/ml for SUDV on Vero cells [16].

As a result of the higher affinity for the GP_1,2_ ectodomain, greater number of contacts, and presumably easier access to its epitope, one might expect KZ52 to neutralize better than 16F6. Surprisingly, however, 16F6 neutralizes SUDV GP_1,2_-bearing VSIV better than KZ52 neutralizes EBOV GP_1,2_-bearing vesicular stomatitis Indiana virus (VSIV) [16] (data replotted here as Figure 9). At all IgG concentrations, a greater portion of virus remains un-neutralized by KZ52 than by 16F6. Even small differences in neutralization capacity may confer significant functional variation *in vivo* for filoviruses because a single p.f.u. can be a lethal dose for primates.

Our data suggests that binding affinity and access to the epitope alone do not account completely for antibody neutralization efficacy; the composition of the particular epitopes and the susceptibility of the different GP_1,2_, themselves, to be neutralized are also important. For example, although the epitopes overlap, KZ52 is shifted more towards GP_2_, burying mostly GP_2_ while 16F6 buries more of GP_1_ on SUDV GP_1,2_ [16]. Affinity for transmembrane-anchored GP_1,2_ could differ somewhat from affinity for the GP_1,2_ ectodomain as well, although the epitopes of these antibodies are quite some distance (>50 Å) from the transmembrane anchor.

In addition, the 16F6 and KZ52 Fabs are rotated relative to each other when bound to GP_1,2_. KZ52 binds with its heavy chain “down” toward the membrane, while 16F6 binds with its heavy chain “up” toward GP_1_. In addition, the crystal structures reveal that the heavy chain of 16F6 interacts directly with both heptad repeat 1 of GP_2_ (specifically HR1_B_) and the β_2_ strand of GP_1_. By contrast, KZ52 interacts primarily with the N-terminal peptide of GP_2_ and the turn region prior to the HR1 helix. KZ52 is unable to make the same contacts as 16F6 because (1) its heavy and light chain are switched in space relative to those of 16F6 and (2) the N-terminal peptide of GP_2_ is nestled into the center of the paratope in EBOV (Figure 7). The N-terminal peptide of GP_2_ is probably less important for GP_1,2_ function than are the HR1 helix and GP_1_ base strand. Hence, an antibody that anchors a less-important terminal peptide might be expected to be less effective than one that anchors a heptad repeat critical for conformational change to its prefusion GP_1_β-sheet clamp. Hence, 16F6 binds more weakly, but may bind more effectively. These antibodies are thought to neutralize by blocking conformational changes during fusion. Perhaps better bridging of GP_1_ and GP_2_ by 16F6 more effectively anchors the pair in a pre-fusion conformation.

Alternatively, key differences between EBOV and SUDV GP_1,2_ may render them differently susceptible to neutralization. GP_2_ of SUDV is less stably ordered than GP_2_ of EBOV. Perhaps 16F6 has recognized a greater portion of GP_1_ because its GP_2_ is a “moving target”. Certainly, the increased mobility of the GP_2_ N-terminal peptide in SUDV means that more of underlying GP_1,2_ structure is available for antibody interaction, antibody anchoring of the prefusion conformation and greater neutralization. Another difference is that EBOV GP_1,2_ has a greater requirement for cathepsin cleavage than does SUDV GP_1,2_ [18], and crystal structures reveal that the two GP_1,2_ have different electrostatics at their trimer interface. Electrostatics at the trimer interface may influence how the low pH environment of the endosomes determines susceptibility of GP_1,2_ to triggering [16].

In summary, we find by structural and functional analysis, that the antibody of higher affinity and presumably easier access to a shared site is not necessarily the most effective at viral neutralization. Other characteristics of the epitope bound, including which amino acids are contained in the individual epitopes and the functional importance of these amino acids, may confer neutralization susceptibility. A conundrum for filoviruses has been that antibody efficacy *in vitro* and *in vivo* do not always correlate. Here, we demonstrate that certain *in vitro* characteristics of antibody binding strength, epitope access, and *in vitro* neutralization also do not necessarily correlate. In addition to binding tightly, an effective antibody may need to bind correctly. 

Development of effective immunotherapeutics against filoviruses will thus require detailed and coupled structural and functional characterization of candidate antibodies. It will also require production and analysis of larger panels of antibodies against the filoviruses than have been currently described so that we may compare several different antibodies within the same competition group, in order to determine what the most effective antibodies are and why this is so. This knowledge base will also clarify what would constitute an ideal antibody response elicited by vaccination, and will aid in development of vaccines designed to elicit these types of antibodies.

**Table 1:** Interactions made by key residues in the “chain reversal region” in pre-fusion and post-fusion forms of ebolavirus GP_1,2._

Residue	Prefusion (SUDV-Bon)	Prefusion (EBOV-May)	Postfusion (EBOV-May)
R587	Hydrogen bond to T60′	No interactions	Hydrogen bond to N586′
F592	Stacks against L57, I185, L594′, R596	Stacks against V48, L57, L593	Stacks against L594′, W597′, I603′,
W597	Stacks against W597′, W597′′	Side chain disordered	Stacks against F592′, L593′, L594, I603, R596
G598	No interactions	No interactions	Hydrogen bond to H602
H602 (R in SUDV)	Side chain disordered	Residue disordered	Hydrogen bond to G598
I610	Side chain disordered	Residue disordered	No interactions

Note that ′ and ′′ denote residues from the other two monomers related by the three-fold axis

Table 2Interactions between SUDV GP_1,2_ and Fab 16F6.

**Table:** GP_1_ to antibody heavy chain*

16F6-H			GP1	Interaction
Y31	OH	O	S32	H-bond
Y31	OH	OE2	E47	H-bond
Y32	OH	OE1	E44	H-bond
R98	NH2	OE1	E44	H-bond
Y31	OH	C	S32	Nonbonded
Y31	CE1	CG	P34	Nonbonded
R98	NH2	CD	E44	Nonbonded
R98	NH2	OE2	E44	Nonbonded
Y32	OH	N	V45	Nonbonded
A28	CB	O	V45	Nonbonded
Y31	OH	CG	E47	Nonbonded

**Table:** GP_2_ to antibody heavy chain

16F6-H			GP_2_	Interaction
N103	ND2	O	A553	H-bond
Y101	OH	OE1	E564	H-bond
Y101	OH	OE2	E564	H-bond
Y101	O	CA	G557	Nonbonded
Y101	CE1	CB	Q560	Nonbonded
Y101	CE1	N	L561	Nonbonded
Y101	OH	CA	L561	Nonbonded
Y101	OH	CD	E564	Nonbonded
Y101	OH	N	L561	Nonbonded

**Table:** GP_1_ to antibody light chain

16F6-L			GP_1_	Interaction
T56	CG2	ND2	N40	Nonbonded

**Table:** GP_2_ to antibody light chain

16F6-L			GP_2_	Interaction
W50	NE1	C	N552	Nonbonded
W50	CD2	CB	N552	Nonbonded
W50	NE1	CB	N552	Nonbonded
W50	CE2	CB	N552	Nonbonded
W50	CD1	N	N553	Nonbonded

*Interactions were calculated with the PDBsum server and confirmed by visual inspection with COOT. The cutoff distance for non-bonded interactions listed above is 3.5 Å.

**Table 3:** Comparison of the 16F6 and KZ52 epitopes on SUDV and EBOV GP_1,2_, respectively. Residues at the shared intersection of the two epitopes are colored orange. Residues listed are conserved between SUDV and EBOV, unless specified.

Residues of SUDV GP_1,2_ bound by 16F6	Residues of EBOV GP_1,2_ bound by KZ52
S32	
P34	
T39 (note: residue 39 is H in EBOV)	
N40	
	S41
T42	V42
L43	L43
E44	Q44
V45	
T46 (is S in EBOV)	
E47 (is D in EBOV)	
Q50 (is K in EBOV)	
V52	
	A503 (is T in SUDV)
	I504 (is N in SUDV)
	V505 (is T in SUDV)
	N506 (is K in SUDV)
	A507
	Q508 (is T in SUDV)
	P509 (is G in SUDV)
	K510
	C511
P513	P513
	N514
	H549
N550	N550
Q551	Q551
N552	D552
A553	G553
C556	C556
G557	
Q560	
L561	
E564	

**Table 4:** Data collection and refinement statistics of SUDV-Bon GP_1,2_ bound to 16F6.

Beamline	ALS 8.3.1
Space group	I23
Cell parameters (Å)	a=b=c=194.86
Wavelength (Å)	1.1159
Resolution (Å) (last shell)	50.00-3.35 (3.47-3.35)^a^
Total observations	149425
Unique reflections	17818
I/σ	13.4 (2.8)
Rsym (%)^b^	7.9 (77.7)
Completeness (%)	99.9 (100.0)
Redundancy	8.4
Matthews coefficient (V_M_, A^3^Da^-1^)	3.5, 65% solvent content
	
R_work _(%)^c^	21.98
R_free_ (%)	28.44
Number of protein residues/atoms	782/6003
Number of glycan residues/atoms	9/111
r.m.s.d. bond length (Å)	0.012
r.m.s.d. bond angles (°)	1.561
Average B values (Å^2^)	
Fab 16F6 (chains heavy/light)	116/124
GP_1_	175
GP_2_	148
Overall	141
Ramachandran plot (%)	
Most favored	78.8
Allowed	18.4
Generously allowed	2.2
Disallowed	0.6

^a^Values in parenthesis are for the highest resolution shell. ^b^R_sym_ = ΣΣ_i_| I_i_ - <I> | /Σ<I>, where <I> is the mean intensity of the N reflections with intensities I_i_ and common indices h,k,l. ^c^R factor = Σ_hkl_||F_obs_|-k|F_cal_|/Σ_hkl_|F_obs_|, where F_obs_ and F_cal_ are observed and calculated structure factors respectively.

## 3. Experimental Section

### 3.1. Molecular Biology

Sudan virus Boniface (SUDV-Bon) glycoprotein (GP_1,2_) was cloned into the pDISPLAY vector (Invitrogen) for expression in mammalian cells with an Ig K-chain leader sequence in place of its natural signal sequence, and a Factor Xa-cleavable N-terminal HA tag for purification. The construct includes residues 33-313 and 473-637, with deletion of the mucin-like domain (residues 314–472) and C-terminal transmembrane domain (residues 638–676). The GP_1,2_ gene was amplified by PCR using oligonucleotides containing BglII restriction sites at their 5′ ends and SalI restriction sites at the 3′ end. The PCR fragment was then cloned into pDISPLAY using BglII and SalI DNA restriction and T4 ligase DNA ligation to produce the IgGK secretion leader-HA-FactorXa-ARS-GPdelta mucin deltaTM plasmid. GP_1,2_ is composed of more than 50% oligosaccharides by weight, primarily because of a heavily glycosylated mucin-like domain that is non-essential for cellular attachment or entry [24].

### 3.2. Protein Expression

Recombinant SUDV GP_1,2_ was transiently expressed in HEK293T cells by calcium phosphate precipitation in ten-layer CellStacks (Corning). The DNA–calcium phosphate mixture was added to 70% confluent cells grown in DMEM plus 1x penicillin-streptomycin and 5% (v/v) fetal bovine serum. The supernatant was harvested four days post-transfection, concentrated using a Centramate tangential flow system and purified by anti-HA immunoaffinity chromatography. GP_1,2_ was natively deglycosylated using Peptide:N-glycosidase F (PNGaseF) in PBS, overnight at room temperature. The extent of the digestion was followed by SDS-PAGE.

### 3.3. Structural Determination of SUDV GP–Fab 16F6 Complex.

For crystallization, deglycosylated SUDV GP_1,2_ was mixed with excess Fab 16F6 and incubated for 1 hour at 4° C and subequently purified on a Superdex-200GL 10/300 column equilibrated with 10 mM Tris-HCl, pH 7.5 and 150 mM NaCl. Fractions containing trimeric SUDV GP_1,2_-Fab16F6 complex were concentrated to 10 mg/ml and initial crystallization conditions were screened using a Topaz Fluidigm system at 20° C. Initial crystal hits were obtained in the presence of 20% PEG 3350 and 0.2 M lithium citrate and were translated and optimized by hanging-drop vapor diffusion. Initial crystals grew over a period of one month at room temperature up to a maximum size of 0.2 × 0.2 × 0.2 mm^3^ and have a cubic morphology. Initial crystals diffracted up to 4.5 Å at the Beamline 8.2.2 of the Advanced Light Source (ALS, Berkeley). Improved crystals were obtained by adding a mixture of 3 μL of the SUDV GP_1,2_-Fab16F6 complex at 10 mg/mL in 10 mM Tris HCl pH 7.5 and 0.5 μL of 20% benzamidine hydrochloride to 3 μL of mother liquor crystallization solution 15% (w/v) PEG 3350, and 0.2 M lithium citrate. These crystals grew in 3-4 weeks at room temperature to a maximum size of 0.2 × 0.3 × 0.3 mm^3^ and have a star-shaped morphology. The crystals were cryoprotected with 17.5% (v/v) glycerol plus mother liquor before flash cooling in liquid nitrogen. The crystals diffracted beyond 3.2 Å resolution, but due to radiation damage, a complete dataset was collected with good statistics up to 3.35 Å, at ALS beamline 8.3.1. The data were indexed, integrated and scaled using HKL2000 [25]. The SUDV GP_1,2_–Fab16F6 crystals belong to space group I23, with unit cell dimensions of a=b=c=194 Å. The solvent content is approximately 65% [26] with one monomer of the GP_1,2_Fab complex in the asymmetric unit. Data collection statistics are shown in Table 4.

The structure of SUDV GP_1,2_ was determined by molecular replacement using an edited poly-alanine model based on the sequence alignment and on the conservation of structure elements between SUDV GP_1,2_ and the previously determined EBOV-May GP_1,2_ structure [12]. The edited poly-alanine search model comprised residues 62-113, 129-186, 215-260 from GP_1_ and 503-595 from GP_2_. An initial molecular replacement solution was found using the first 4.8 Å data set using PHASER [26]. A partial solution for the glycoprotein model was obtained and revealed unambiguous extra electron density for key parts that were absent from the search model. This partial solution was fixed and the Fab position was determined using poly-alanine models of separate variable and constant domains of a different Fab (PDB 1JLP) [27], with CDRs deleted. Interpretable electron density maps with clear secondary structural elements and solvent boundaries were obtained, with the biologically relevant trimer generated by the crystallographic three-fold axis that extends through the diagonal vertices of the cubic spacegroup I23. This initial solution was assembled into a complete search model and used with the subsequent 3.35 Å data set in PHASER (LL-gain=604, TFZ=33.8, RFZ=10.4). After an initial step of rigid body refinement, the structure was refined with CNS [28] using torsion-angle simulated-annealing refinement with a maximum-likelihood amplitude target. The initial model was iteratively improved by visual inspection and model building using COOT [29] and refinement in CNS [28]. In the final rounds of refinement, riding hydrogens, grouped atomic displacement and TLS parameters were implemented using PHENIX [30]. The structure was refined to final R_work _and R_free_ values of 21.98% and 28.44% and contains SUDV-Bon GP_1,2_ residues 32–191, 213–285, 300–311 and 510–614, and Fab 16F6 residues 1–220 (heavy chain) and 1–212 (light chain). Electron density was missing for GP_1,2_ residues 192–212, 286-299, 312–313, 473–509 and 615–637. Weak or discontinuous electron density is observed in the outer regions of the glycan cap (residues 261–285 and 300–307) and these regions were tentatively assigned as poly-alanine fragments. Despite the treatment of the GP_1,2_ with PNGaseF, two glycan chains resistant to deglycosylation at N257 on GP_1_ and N563 on GP_2_ were preserved. These glycans show a high B-factor (B_average_= 242.5 Å^2^), but there is unambiguous electron density for the chitobiose core and up to the first mannose residues of these glycans. Final refinement statistics and analysis are shown in Table 4. Atomic coordinates and structure factors are deposited in the RCSB Protein Data Bank under accession number 3VE0. The biologically relevant trimer was generated by the crystallographic three-fold axis that extends through the diagonal vertices of the cubic spacegroup I23.

### 3.4. Antibody binding studies

The binding affinity of KZ52 to EBOV-May GP_1,2_ and 16F6 to SUDV-Gul and -Bon GP_1,2_ was measured using BIAcore-2000 Surface Plasmon Resonance spectrophotomer. All the samples were buffer exchanged to HBS-EP buffer (10 mM Hepes, pH 7.4, 150 mM Sodium Chloride, 3 mM EDTA, and 0.005% Polysorbate 20) prior to complexation. KZ52 and 16F6 were immobilized at 25 °C on various channels of carboxymethylated dextran CM5 sensor chips to ~200 response units using standard amine coupling protocol. EBOV GP_1,2_, SUDV-Bon GP_1,2_ and SUDV-Gul GP_1,2_ were injected over channels coated with KZ52 and 16F6 respectively. The glycoproteins were injected sequentially at increasing concentrations of 1, 2.5, 5, 10 and 20 µM respectively at a flow rate of 10 µL/min for 250 sec and dissociation of the complexes were measured over a period of ~600 sec. The chromatogram data were analyzed using a 1:1 Langmuir binding model to determine the k_a_ (on), k_d_ (off) and K_d_ (dissociation) values using the BIAevaluation software.

### 3.5. Antibody Neutralization Studies

VSIV pseudotypes bearing EBOV or SUDV GP and expressing eGFP were generated and concentrated by ultracentrifugation as described previously [31,32]. Virus-antibody incubations were carried out as follows: Concentrated virus (1-3 µL, ~1×10^5^ IU) was mixed with PBS containing antibody at the indicated concentration (0-40 µg/mL final; total reaction volume, 20 µL). Reactions were incubated at room temperature for 1 hour, and viral infectivity was determined by titration on Vero cell monolayers. Briefly, Vero cells were exposed to samples for 1 hour at 37° C and infectivity was scored at 16 hours post-infection by manually enumerating GFP-positive cells, as described previously [32].

### 3.6. Deuterium Exchange Mass Spectrometry Experiments

A detailed description of DXMS methodology was described previously [17]. Briefly, quench conditions that produced an optimal pepsin fragmentation pattern were established for both EBOV and SUDV GP1,2. Then, the non-deuterated samples, functional deuterated samples and the equilibrium-deuterated back-exchange control samples were prepared by mixing the GP_1,2_ proteins with non-deuterated (8.3 mM Tris, 150 mM NaCl, in H_2_O, pH 7.15), deuterated (8.3 mM Tris, 150 mM NaCl, in D_2_O, pD_READ_ 7.2) and equilibrium-deuterated (1% formic acid in 99.9% D_2_O) buffers, separately. Later, optimized quench buffer was added to stop the reactions. Finally, the samples were passed over pepsin column, and the resulting peptides were collected on a C18 trap and separated using a C18 reversed phase column. Data were acquired in both data-dependent MS/MS mode and MS1 profile mode by a LCQ mass spectrometer, and the data analyzed by SEQUEST (Thermo Finnigan Inc.) and DXMS explorer (Sierra Analytics Inc., Modesto, CA).

## 4. Conclusions

We report the crystal structure of SUDV-Bon GP_1,2_ bound to 16F6 and describe new structural details of the complex including thermal motion of GP_2_, approach angle and access to the antibody epitope and molecular interactions between antibody and GP_1,2_. Surface Plasmon Resonance studies, also presented here, reveal that 16F6 binds SUDV-Gul/Bon GP_1,2_ with a lower affinity than KZ52 binding to EBOV-May GP_1,2_. Paradoxically, however, 16F6 confers better neutralization than KZ52. Together, these studies suggest that the key to effective neutralization might lie in binding the epitope correctly rather than in affinity alone.
